# Unstable Chromosome Aberrations Do Not Accumulate in Normal Human Fibroblast after Fractionated X-Irradiation

**DOI:** 10.1371/journal.pone.0116645

**Published:** 2015-02-27

**Authors:** Mitsuaki Ojima, Maki Ito, Keiji Suzuki, Michiaki Kai

**Affiliations:** 1 Department of Environmental Health Science, Oita University of Nursing and Health Sciences, 2944-9 Megusuno, Oita 840-1201, Japan; 2 Department of Radiation Medical Sciences, Atomic Bomb Disease Institute, Nagasaki University, 1-12-4 Sakamoto, Nagasaki 852-8523, Japan; ENEA, ITALY

## Abstract

We determined the frequencies of dicentric chromosomes per cell in non-dividing confluent normal human fibroblasts (MRC-5) irradiated with a single 1 Gy dose or a fractionated 1 Gy dose (10X0.1 Gy, 5X0.2 Gy, and 2X0.5 Gy). The interval between fractions was between 1 min to 1440 min. After the completion of X-irradiation, the cells were incubated for 24 hours before re-plating at a low density. Then, demecolcine was administrated at 6 hours, and the first mitotic cells were collected for 42 hours. Our study demonstrated that frequencies of dicentric chromosomes in cells irradiated with a 1 Gy dose at different fractions were significantly reduced if the fraction interval was increased from 1 min to 5 min (p<0.05, χ^2^-test). Further increasing the fraction interval from 5 up to 1440 min did not significantly affect the frequency of dicentric chromosomes. Since misrejoining of two independent chromosome breaks introduced in close proximity gives rise to dicentric chromosome, our results indicated that such circumstances might be quite infrequent in cells exposed to fractionated X-irradiation with prolonged fraction intervals. Our findings should contribute to improve current estimation of cancer risk from chronic low-dose-rate exposure, or intermittent exposure of low-dose radiation by medical exposure.

## Introduction

Exposure to ionizing radiation (IR) induces multiple types of DNA damage—including base and sugar damage, single-strand breaks, double-strand breaks (DSBs), and cross-links involving DNA and proteins. Among various types of damage, DSBs are the most deleterious, which result in cell death and chromosome aberrations [1–4]. DSBs trigger structural alterations in higher-order chromatin structure, which activates ATM protein encoded by the *ataxia telangiectasia mutated* (*ATM*) gene. Activated ATM by auto-phosphorylation stimulates phosphorylation of an array of downstream targets, including the tumor suppressor protein p53, which regulates DNA damage response (DDR). DDR stimulates DNA repair, and simultaneously, it arrests cell cycle while repair is ongoing. DSBs can be repaired by two major repair processes: 1) homologous recombination, which requires an undamaged sister chromatid DNA strand as a template for repair replication or 2) nonhomologous end-joining, which is actually end-to-end rejoining that follows “trimming” of the ends of the breaks [5, 6]. Homologous recombination has been thought as a highly precise and accurate process [7], while nonhomologous end-joining is relatively inaccurate and sometimes leads to chromosomal deletions, insertions, and translocations [8, 9]. Such chromosome aberrations cause large-scale changes to genomic sequences in the progeny of surviving cells, so that genomic instability, a hallmark of carcinogenesis, is induced. The relationship between acute exposure to IR and chromosome aberrations has been investigated extensively, among which a dicentric chromosome has been treated as the most established marker. Dicentric chromosomes display a linear-quadratic, dose-response relationship after acute IR exposure [10–26], however, the dose-response could be different depending on dose-rate. Consequently, chromosome aberration may not be an adequate biomarker to evaluate the risks associated with chronic exposure to radiation. The hit theory of radiation biology predicts that the linear portion of the dose-response curve predominates at low-dose-rates and the linear portion is a limiting slope at low doses [12, 15, 18]. Therefore, for a given IR dose, low-dose-rates are less effective than high-dose-rates for inducing dicentric chromosomes, and this difference in effect is called the dose-rate effect (DRE). The DRE has been provided in several biological end points including the induction of mutation and oncogenic transformation [27–31]. It is well recognized that the DRE is a key factor in estimating radiation-related cancer risk. However, a dose-rate dependency of DRE has not been sufficiently elucidated yet.

Here, we examined the dose-rate effects by using the fractionated dose per unit time. We have hypothesized if the initial yields of DSBs per unit time were reduced as fractionating dose per unit time, then the DSBs are being repaired correctly and efficiently. Thus, the induction of dicentric chromosomes could be suppressed by reducing the dose per unit time, in other words, the fraction intervals.

To test the possibility, we determined the frequencies of dicentric chromosomes in normal human fibroblasts (MRC-5 cells) that had been subjected to fractionated X-irradiation. We found that frequencies of dicentric chromosomes in cells irradiated with a single 1 Gy dose were similar to those in cells irradiated with a fractionated 1 Gy dose (10×0.1 Gy, 5×0.2 Gy, and 2×0.5 Gy) when the fraction interval was 1 min. Notably, the frequency of dicentric chromosomes per cell decreased in cells subject to fractionated X-irradiation when the fraction interval was increased from 1min to 5 min, while the frequencies of dicentric chromosomes were constant for all fraction intervals longer than five minutes.

Based upon these findings, we conclude that the fraction interval is the critical factor affecting the generation of dicentric chromosomes, and longer fraction intervals, corresponding to low-dose-rates, are likely to serve correct DSB repair. In contrast, shorter fraction intervals tend to create more dicentric chromosomes, indicating that high-dose-rate exposure is more harmful to the integrity of the genome. These results should provide mechanistic insights into our understandings of the DRE.

## Materials and Methods

### Cell Culture

MRC-5 cells (European Collection of Cell Cultures), primary normal fibroblasts from the human lung, were grown in T-25 tissue culture flasks in Minimum Essential Medium Eagle (MEM) with Earle’s salts, L-glutamine and NaHCO_3_ that was supplemented with 10% fetal bovine serum and penicillin-streptomycin. Cells were maintained in a humidified incubator at 37°C with 5% CO_2_. X-irradiation was performed on non-dividing confluent cell cultures.

### X-irradiation

An X-ray generator (HF320, Shimadzu, Tokyo, Japan) was used to deliver all X-rays. The generator was operated at 30 mA and at 90 kV for the delivery of 0.1 Gy or at 18 mA and at 200 kV for the delivery of 0.2, 0.5, or 1 Gy. The quality of X-ray related with kV would not influence the RBE so much since the effect of the quality may be much smaller than of exposure dose [32, 33]. The generator was equipped with a filter system composed of a plate comprising 1-mm copper and 1-mm aluminum for 0.1 Gy or a plate comprising 0.5-mm copper and 0.5-mm aluminum for 0.2 Gy, 0.5 Gy, or 1 Gy. To achieve an irradiation time less than 1 min, dose-rates were set between 0.2∼1 Gy/min depending on the distance from the source. For doses up to 0.1 Gy, a dose-rate of 0.2 Gy/min was used, while for doses up to 1 Gy, a dose-rate of 1 Gy/min was used. An ionization chamber was used to determine each dose-rate. The different dose-rates (0.1∼1 Gy/min) have shown no significant difference in the frequency of dicentric chromosomes [10, 16, 19, 20–24].

**Fig 1:**
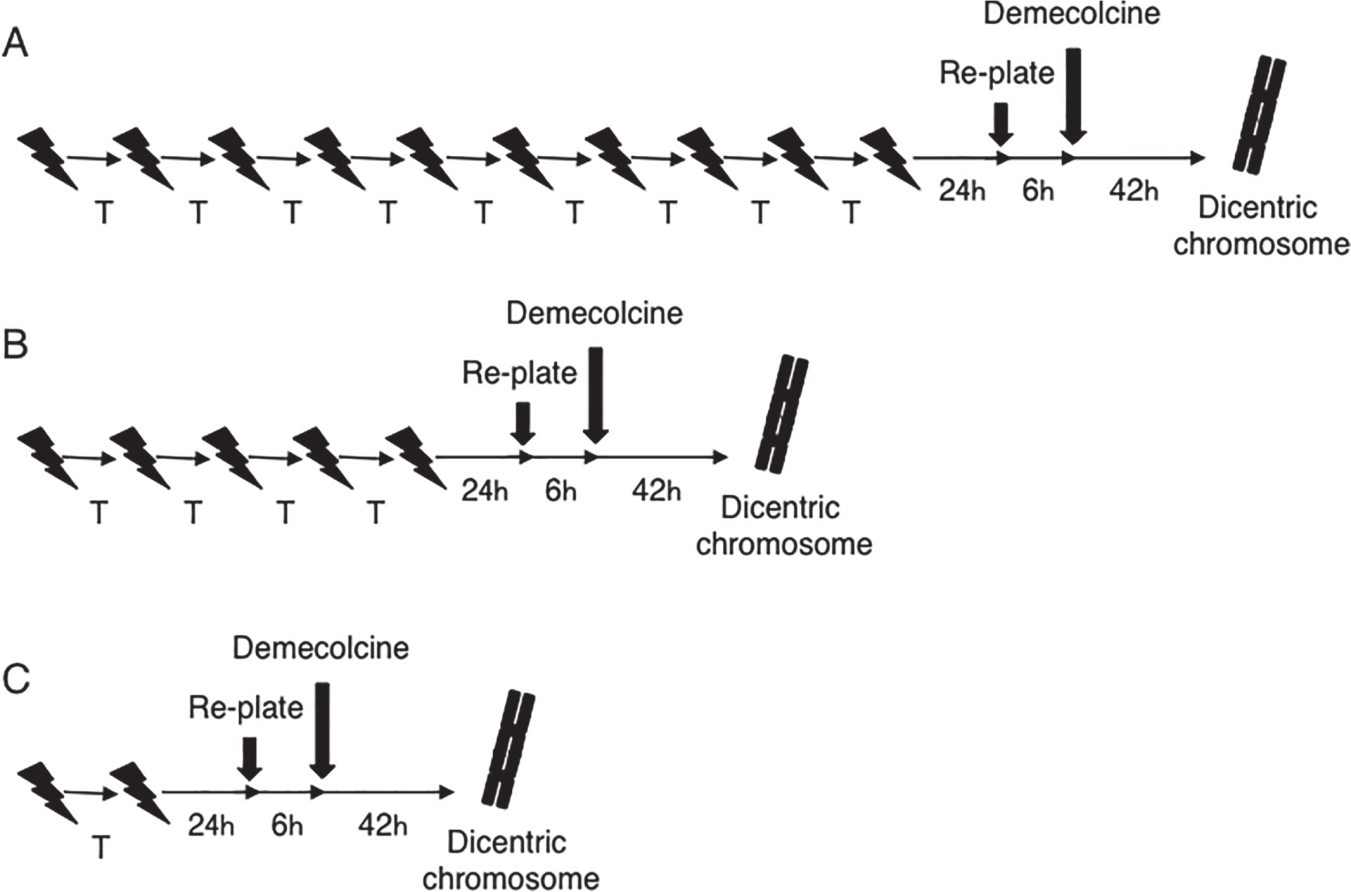
The schemes of fractionated X-irradiation A: 10×0.1 Gy. B: 5×0.2 Gy. C: 2×0.5 Gy. T indicates each time interval between fractions; these intervals ranged from 1 min to 1440 min.

Single-dose (0.1 Gy, 0.2 Gy, 0.5 Gy, or 1 Gy) and fractionated X-irradiation schemes (10×0.1 Gy, 5×0.2 Gy, or 2×0.5 Gy) were performed. For fractionated X-irradiation, the fraction interval was set between 1 min and 1440 min (Fig. 1A-C). We have already performed radiation dosimetry of dose by fractionated X-irradiation, and have checked that the doses did not change by fractionated X-irradiation of a short time interval. In this study, sham-irradiated cells served as controls.

### Conventional giemsa staining method

Conventional Giemsa staining was used to detect dicentric chromosomes. In brief, for each experiment, confluent cells in two T-25 tissue culture flasks were irradiated with X-rays. Cells were then harvested 24 hours after X-irradiation from each T-25 tissue culture flask and mixed together. Mixed cells from two flasks were re-seeded into one T-75 tissue culture flask and grown for 6 hours; demecolcine was added at a final concentration of 20 ng/ml, then each flask was incubated for an additional 42 hours to allow cells to accumulate in metaphase of the first cell division. We have investigated the cytotoxic effects of 20 ng/ml demecolcine for 48 hours. Propidium iodide staining revealed 8% dead cells in a sample of >400000 cells threated with 20 ng/ml demecolcine for 48 hours. A control population of 2.7 million cells incubated 48 hours without treatment contained 9% dead cells. Thus, we have concluded that the experimental condition for the metaphase arrest is not cytotoxic to cells. Mitotic cells were then collected by tapping the flasks to dislodge any metaphase cells. Mitotic cells were incubated in a hypotonic solution (0.075 M KCl in H_2_O) for 20 min, and then the swollen cells were fixed with Carnoy’s fixative (methanol and acetic acid = 3:1). Each suspension of fixed cells was stored at −20°C until use. Mitotic cells were dropped onto pre-cleaned glass microscope slides and allowed to dry for 1 day at room temperature. Each glass slide with metaphase chromosomes was dipped in 5% Giemsa solution and incubated for 10 min at room temperature. Each slide was then washed in distilled H_2_O and dried with a hair dryer. Metaphase chromosomes were viewed under a microscope. We scored the frequencies of dicentric chromosomes per cell.

### Detection of mitotic cells by immunocytochemistry

**Fig 2:**
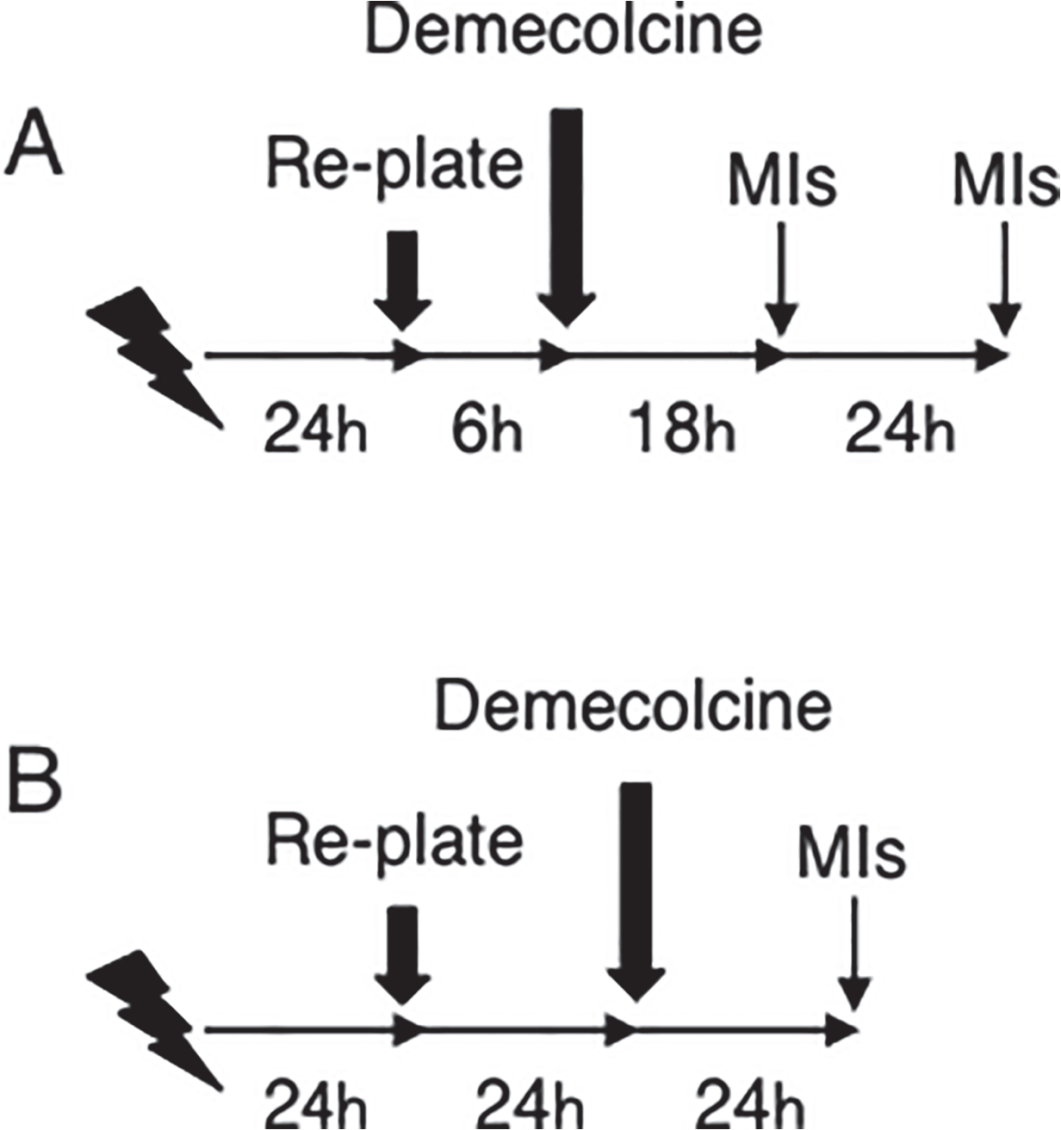
The schemes to detect the frequency of mitotic MRC-5 cells. A: Examination of the mitotic indices (MIs) during the first and the second 24 hour-incubations after the replating. B: Examination of the MIs between 24 hours and 48 hours after the replating.

Cells were harvested 24 hours after X-irradiation from T-25 tissue culture flask. 1×10^5^ cells were re-seeded onto 20×20 mm cover-glass in 35 mm dish and grown for 6 hours; demecolcine was added at a final concentration of 20 ng/ml, then cells were incubated for an additional 18 hours or for an additional 42 hours (Fig. 2). Cells were fixed with cold methanol for 10 minutes on ice, washed with phosphate-buffered saline (PBS) three times, and stored at 4°C. The primary antibody (anti-phosphorylated histone H3 at serine 10, clone 3H10, Millipore Japan, Tokyo) was diluted in 100 μl of TBS-DT (20 mM Tris-HCl, 137 mM NaCl, pH7.6, containing 50 mg/ml skim milk and 0.1% Tween-20), applied on the samples, and the samples were incubated for 2 hours in a humidified CO_2_ incubator at 37°C. Then, the primary antibody was washed with PBS, and Alexa488-labelled Goat anti-mouse IgG antibody (Molecular Probes, Inc., OR) was added. The samples were incubated for another 1 hour in a humidified CO_2_ incubator at 37°C, washed with PBS and counterstained with 0.1 mg/ml of DAPI.

The samples were examined with a widefield microscopy equipped with motorized stages (F3000B fluorescence microscope, Leica, Tokyo). Digital images were captured and the images were analyzed by FW4000 software (Leica, Tokyo).

### Statistical analysis

Statistical significance was evaluated by χ^2^-test, and we considered p<0.05 to be statistically significant.

## Results

### Frequency of the 1st mitotic cells during the 48 hours after re-plating

We used the histone H3 immunofluorescence foci assay to investigate the mitotic indices (MIs) during the first and the second 24 hour-incubations after the replating, and then compared the MIs with those observed in cells incubated for 48 hours after the replating. The results showed that the MIs for 48 hour-incubations were approximately 10% higher than the MIs at 48 hour, both for control and irradiated cells. The difference between 24 and 48 hour was also around 10% of the results from the first 24 hour-incubations (Table 1). This effect was due to more cells entering the 1st mitosis, rather than a few coming to their 2nd mitosis, as shown by the fact that no consistent increase of cell number was observed when incubating with demecolcine (Table 2).

**Table 1:** Frequency of mitotic MRC-5 cells

Dose (Gy)	Culture time (hours)	Number of counted cells	Total number of mitotic cells	Mitotic indices
0	24	13604	1002	7.83 ± 2.27
	48	11430	961	8.43 ± 2.66
	24–48	13665	103	0.75 ± 0.31
1	24	12045	645	5.43 ± 1.54
	48	14298	858	6.3 ± 2.09

Mitotic indices were the mean ± standard error of those calculated in 50 randomly selected areas.

**Table 2:** Number of MRC-5 cells after X-irradiation

Dose (Gy)	Culture time (hours)	Number of counted cells	Relative cell number
0	24	190012	1.00
	48	211056	1.11
1	24	179426	1.00
	48	171794	0.96

1×105 cells were plated per 22×22 mm coverslip. Number of cells on three cover slips was calculated by scanning the entire area of each cover slip.

### The frequency of dicentric chromosomes caused by single-dose X-irradiation

**Fig 3:**
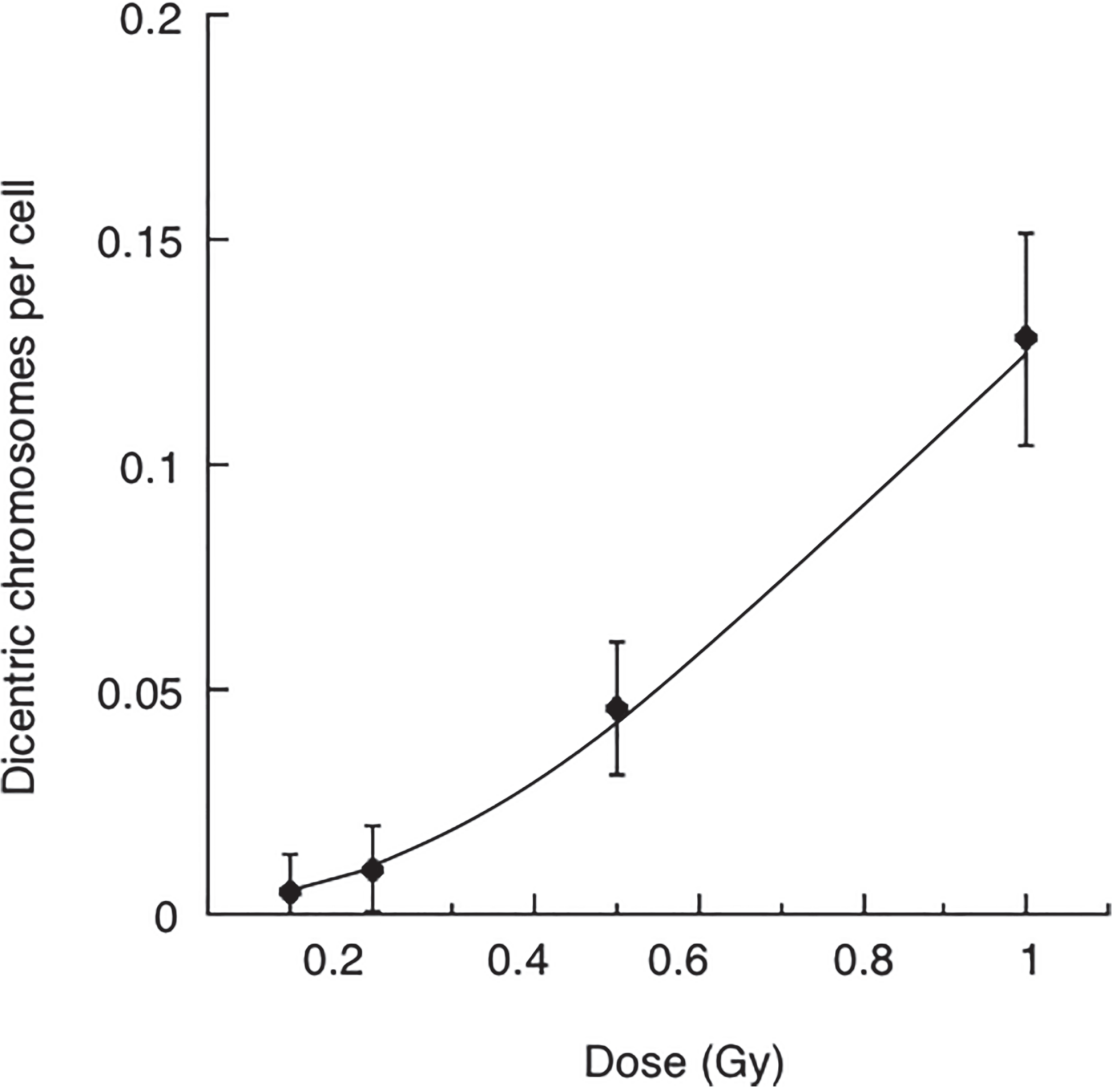
The dose-response of dicentric chromosomes in MRC-5 cells after a single-dose of X-ray irradiation. Full circles: The numbers of dicentric chromosome corrected for the baseline number. Results are means of two or three independent experiments ± standard error.

The observed frequencies of dicentric chromosomes after single-dose X-irradiation of MRC5 cells are shown in Table 3, and the dose-response relationship is shown in Fig. 3. The frequency of dicentric chromosomes in sham-irradiated controls was 0.0081 per cell. In contrast, the frequencies of dicentric chromosomes in X-irradiated cells with 0.1 Gy, 0.2 Gy, 0.5 Gy, or 1 Gy, were approximately 0.0047, 0.0101, 0.0461, and 0.1279 after the subtraction of the frequency observed in the control cells, respectively. The frequencies of dicentric chromosomes increased exponentially when cells were irradiated with doses higher than 0.2 Gy.

**Table 3:** Frequency of dicentric chromosomes in MRC-5 cells after X-irradiation

Dose (Gy)	Number of counted cells	Total number of dicentric chromosomes	Number of dicentric chromosomes per cell	Standard Error
0	248	2	0.0081	0.0057
0.1	312	4	0.0128	0.0064
0.2	330	6	0.0182	0.0074
0.5	277	15	0.0542	0.0136
1	250	34	0.1360	0.0232

### The frequencies of dicentric chromosomes caused by fractionated X-irradiation

**Fig 4:**
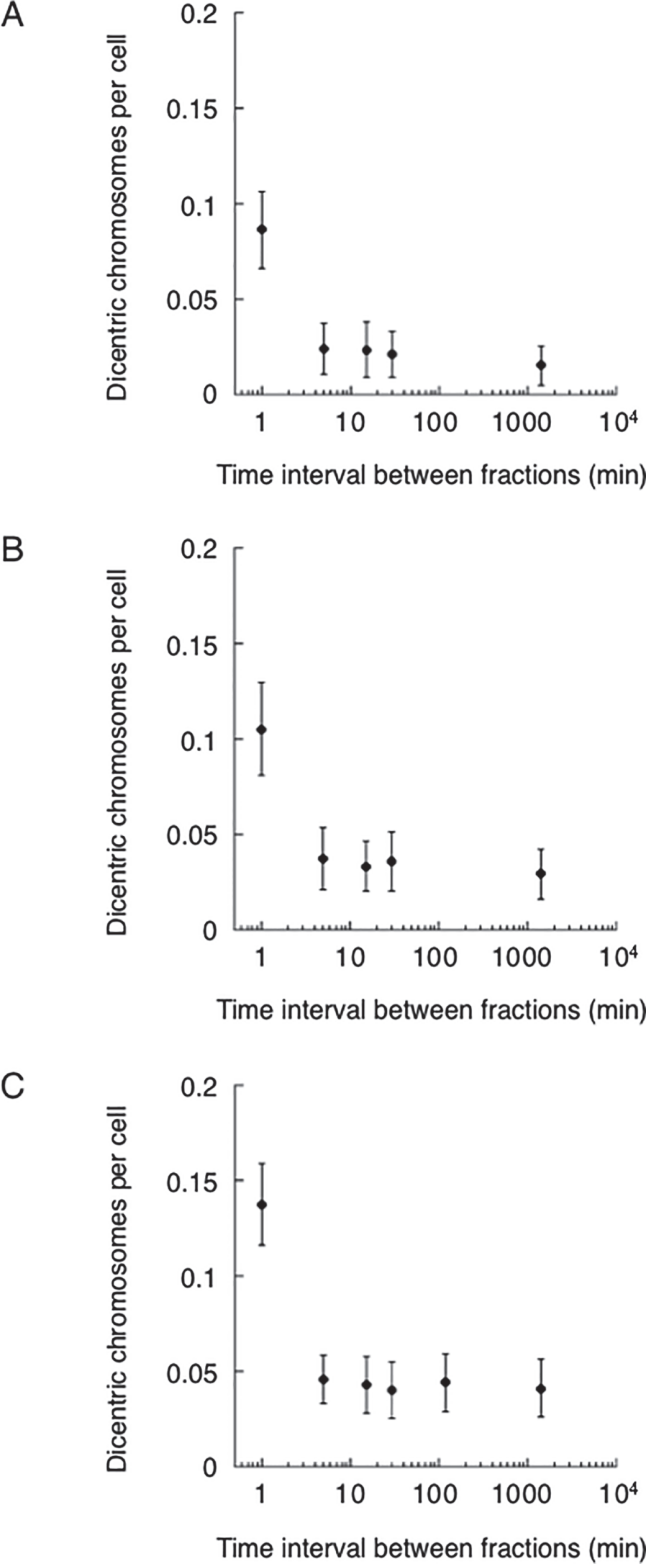
Frequencies of dicentric chromosomes in MRC-5 cells exposed to fractionated X-irradiation. A: 10×0.1 Gy. B: 5×0.2 Gy. C: 2×0.5 Gy. Full circles: The numbers of dicentric chromosome corrected for the baseline number. Results are means of two or three independent experiments ± standard error.

Tables 4 through 6 and Fig. 4 show the frequencies of dicentric chromosomes in MRC-5 cells after the fractionated X-irradiation. With a fraction interval of 1 min and a final dose of 1 Gy, the corrected frequencies of dicentric chromosomes per cell were approximately 0.0863 (Table 4 and Fig. 4A), 0.1052 (Table 5 and Fig. 4B), and 0.1377 (Table 6 and Fig. 4C) for dose fractionations of 10×0.1 Gy, 5×0.2 Gy, or 2×0.5 Gy, respectively. There was no significant difference between fractionated X-irradiation and single-dose of 1 Gy (p>0.05).

**Table 4:** Frequency of dicentric chromosomes in MRC-5 cells after fractionated X-irradiation with 10 × 0.1Gy

Time interval between fractions (min)	Number of counted cells	Total number of dicentric chromosomes	Number of dicentric chromosomes per cell	Standard Error
1	233	22	0.0944	0.0192
5	218	7	0.0321	0.0120
15	222	7	0.0315	0.0134
30	274	8	0.0292	0.0102
24 h	300	7	0.0233	0.0087

**Table 5:** Frequency of dicentric chromosomes in MRC-5 cells after fractioned X-irradiation with 5 x 0.2Gy

Time interval between fractions (min)	Number of counted cells	Total number of dicentric chromosomes	Number of dicentric chromosomes per cell	Standard Error
1	203	23	0.1133	0.0234
5	220	10	0.0455	0.0155
15	289	12	0.0415	0.0118
30	250	11	0.0440	0.0142
24 h	266	10	0.0376	0.0117

**Table 6:** Frequency of dicentric chromosomes in MRC-5 cells after fractioned X-irradiation with 2 × 0.5Gy

Time interval between fractions (min)	Number of counted cells	Total number of dicentric chromosomes	Number of dicentric chromosomes per cell	Standard Error
1	343	50	0.1458	0.0208
5	392	21	0.0536	0.0114
15	254	13	0.0512	0.0139
30	250	12	0.0480	0.0135
120	249	13	0.0522	0.0141
24 h	244	12	0.0492	0.0139

### The effect of increased fraction interval during fractionated X-irradiation

We investigated the effect of extending the fraction interval on the frequency of dicentric chromosome caused by fractionated X-irradiation. With a fraction interval of 5 min and a final dose of 1 Gy, the corrected frequencies of dicentric chromosomes per cell were 0.0240 (Table 4 and Fig. 4A), 0.0374 (Table 5 and Fig. 4B), and 0.0455 (Table 6 and Fig. 4C) for dose fractionations of 10×0.1 Gy, 5×0.2 Gy, or 2×0.5 Gy, respectively, which were significantly lower compared to those obtained by a fraction interval of 1 min. However, further reductions in the frequency of dicentric chromosomes were not observed with fraction intervals longer than 5 min.

## Discussion

Illegitimate rejoining of IR-induced DSBs can lead to chromosome aberrations, such as translocations. While specific translocations are frequently identified in cancer, they are not easy to detect and monitor by conventional cytogenetical techniques. In contrast, dicentric chromosomes can be detected without any difficulty, and previous studies have shown that there is a 1:1 relationship between the incidence of IR-induced dicentric chromosomes and IR-induced translocations [34–36]. Consequently, dicentric chromosomes are often used as alternatives of translocations. Here, we examined the frequencies of dicentric chromosomes in MRC-5 cells following different courses of fractionated X-irradiation. We found that fractionated X-irradiation of 10×0.1 Gy, 5×0.2 Gy, and 2×0.5 Gy with a 1 min fraction interval resulted in a dicentric chromosome frequency similar to that obtained by a single 1 Gy dose of X-irradiation (p>0.05), although the frequencies of dicentric chromosomes were slightly lower for the fractionated X-irradiations (10×0.1 Gy and 5×0.2 Gy), than for the acute 1 Gy. The reduction in the frequencies of dicentric chromosomes caused by fractionated doses of less than 0.5 Gy might be attributed to legitimate rejoining of DSB during the intervals between the fractions. Therefore, we investigated the effect of dose fractionation by extending the fraction interval, and found that frequencies of dicentric chromosomes in cells irradiated with a 1 Gy dose at different fractions were significantly reduced if the fraction interval was increased from 1 min to 5 min (Fig. 4, p<0.05). Interestingly, further increases in the fraction interval above 5 min, even increases to intervals of 1440 min, did not cause any further reduction in the frequency of dicentric chromosomes per cell. Apparently, the frequency of dicentric chromosomes per cell was constant for all fraction intervals greater than 5 min (Fig. 4), indicating that maximum DNA repair efficacy has already been reached by the fraction interval of 5 minutes.

Recent studies have shown that one of the earliest steps in the cellular response to DSB is the phosphorylation of ATM [37–44]. Phosphorylated ATM forms discrete foci at DSB sites [40, 42–44] and leads to the activation of several DNA repair proteins [41]. Phosphorylated ATM foci (p-ATM foci) can be visualized by immunofluorescence using antibodies specific for the phosphorylated form of ATM, and p-ATM foci are used as a biomarker for DSBs [40, 42–44]. Previously, we used p-ATM foci to examine the time course of DSB repair in MRC-5 cells after X-irradiation. The background level of p-ATM foci in these cells is about 0.12 per cell and is almost constant for 48 hours. In contrast, the mean numbers of p-ATM foci per cell are approximately 0.24, 1.0, 5.7, and 34 at 3 min after X-irradiation at a dose of 0.0012, 0.02, 0.2, or 1 Gy, respectively, and the mean numbers of foci per cell did not decrease to the background level during 24 hours incubation time after X-irradiation [43]. These results indicate that residual DSBs can persist for 24 hours after X-irradiation. Rothkamm *et al*. used γ-H2AX foci as a biomarker of DSBs in MRC-5 cells. They observed the highest number of foci at 3 min after X-irradiation, and the number of foci was declining with increasing the time after X-irradiation. However, they found that a very small number of foci could persist for many days in a confluent culture [45]. Based on these findings, a small number of DSBs could persist more than 24 hours after X-irradiation. If DSBs persist for 24 hours after IR, fractionated irradiation involving fraction intervals of 24 hours should cause DSBs to accumulate. Recently, Mariotti *et al*. reported the kinetics of γ-H2AX foci levels in normal human primary fibroblasts, AG01522 cells, following split radiation exposure (2×1 Gy). They found that the number of foci induced by split exposure was smaller compared with the single exposure when the second irradiation was delivered within 5 hours from the first exposure [46]. Here, we found that dicentric chromosomes frequencies in MRC-5 cells were similar for 1 Gy delivered acutely and for 1 Gy delivered in 10×0.1 Gy, 5×0.2 Gy, and 2×0.5 Gy that were 1 min apart; however, when the fraction interval was increased, significantly lower frequencies of dicentric chromosome were observed (p<0.05). Thus, the formation of dicentric chromosomes by fractionated X-irradiation did probably not result from residual DSBs in the intervals between fractions. We assumed that the residual p-ATM foci and γ-H2AX foci might be associated with illegitimate rejoining of DSBs. This idea was supported by a report showing that γ-H2AX foci can localize at the fusion point of dicentric chromosomes [47].

The radioadaptive response represents an important phenomenon that has a significant impact on biological responses induced by radiation. The radioadaptive response is defined as the induction of resistance to subsequent higher doses of radiation by administering earlier, lower doses of radiation [48, 49]. Therefore, the radioadaptive response may constitute a protective effect of low dose radiation. However, an interval of at least 4 hours will be required between the priming dose and challenging dose of IR for full induction of a radioadaptive response [50–52]. Furthermore, the reported priming doses were within the range of 0.003 Gy to 0.2 Gy [50–55]. This study demonstrates that reduced frequencies of dicentric chromosomes were evident under conditions when a radioadaptive response may not have had time to appear. Thus, the effect of fractionated X-irradiation could be independent of the radioadaptive response.

Recently, Tanaka *et al*. reported that, with a low-dose-rate of 0.02 Gy/day, the number of dicentric chromosome in spleen cells increases almost linearly as the total accumulated IR dose increased up to the highest dose of 8 Gy; however, such an increase is not evident with a dose-rate of 0.001 Gy/day [25]. Although the dose-rate was not similar to our study, we are able to assume that mis-joining of DSBs on two different chromosome might have spatial as well as temporal limitations, and this is the reason why accumulation of dicentric chromosome was not observed following fractionated irradiation with prolonged fraction intervals with 5 minutes or more. Our results might provide the biological evidence leading to improve the current cancer-risk estimate following chronic exposure at low-dose-rate. To obtain mechanistic insights at molecular level further studies will be needed to determine a fine-scale energy distribution of radiation deposited in inhomogeneous regions of chromosome [56, 57].
